# Supplementary material for: Faecalibacterium prausnitzii, depleted in the Parkinson’s disease microbiome, improves motor deficits in α-synuclein overexpressing mice
Source: NPJ Parkinsons Dis. 2026 Mar 5;12:94. doi: 10.1038/s41531-026-01287-x (PMC13077053; doi:10.1038/s41531-026-01287-x)
Supplement: Supplementary file 1 — Supplementary_Information [file 41531_2026_1287_MOESM1_ESM.pdf]

SUPPLEMENTARY INFORMATION

for

Faecalibacterium prausnitzii, depleted in the Parkinson's disease microbiome, improves motor deficits in α-synuclein overexpressing mice

Anastasiya Moiseyenko *et al.*

This file includes:

- Supplementary Figures 1 through 11
- Supplementary Tables 1 and 2

Other supplementary materials provided in separate files:

- Supplementary Data

Supplementary Figure 1

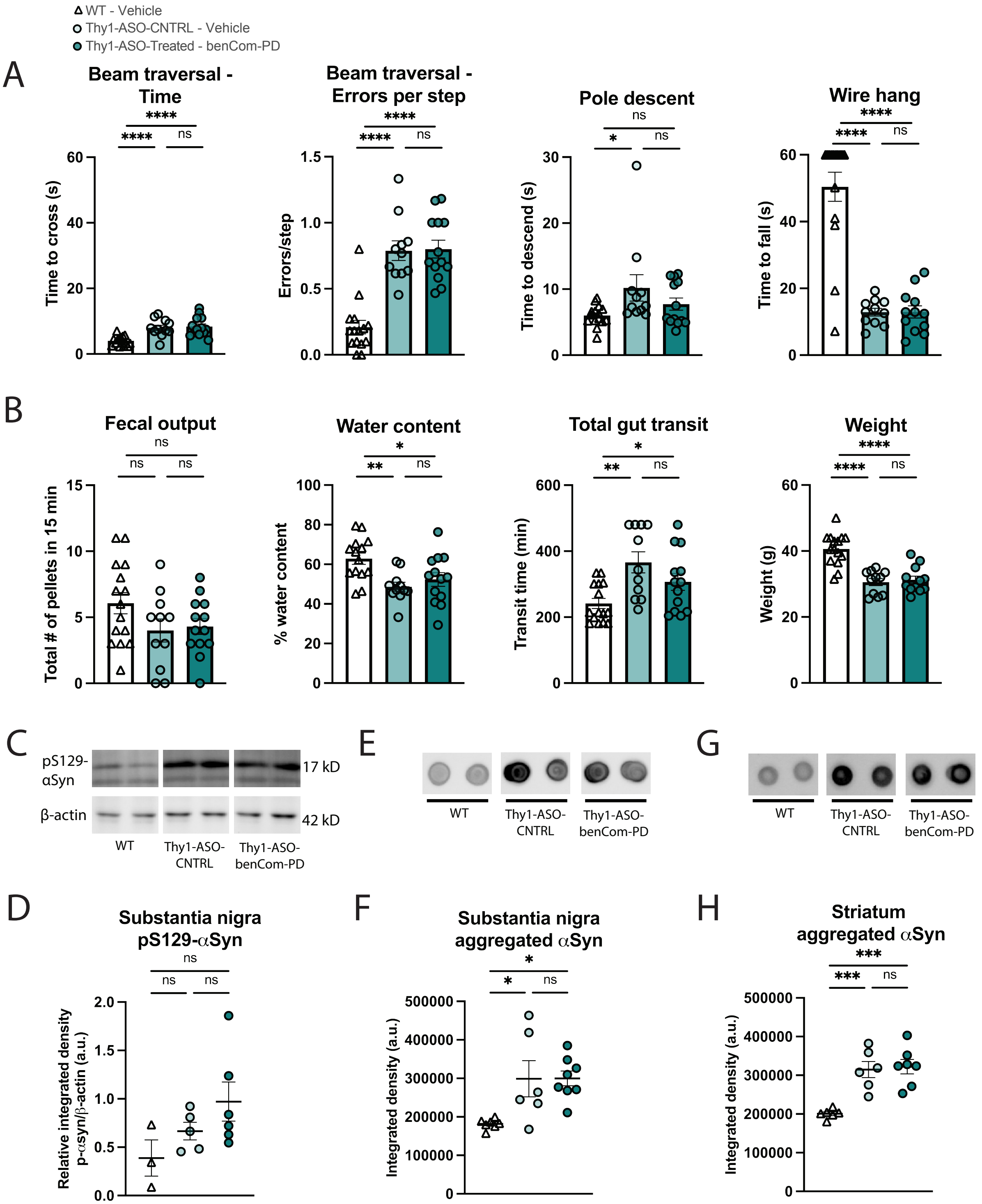

Supplementary Figure 1. **Motor and GI function tests and α-synuclein assays not altered by benCom-PD treatment.** *(Associated with Figure 1)*. (**A**) Motor function and (**B**) GI function

and animal weights of benCom-PD-treated Thy1-ASO animals and controls tested at 20 weeks. n = 11-15. Points represent individual animals; bars represent standard error of the mean. (**C**) Representative images of Western blots of phosphorylated-S129-α-synuclein in substantia nigra and (**D**) quantification. n = 3-6. (**E-H**) Representative images of dot blots for aggregated α-synuclein in (E) substantia nigra and (G) striatum and (F, H) quantification. n = 6-8. Points represent individual animals; bars represent standard error of the mean. See Supplementary Fig. 10 for uncropped blot images. Behavior data in (A, B) analyzed by Kruskal-Wallis test followed by the Conover-Iman post-hoc test with Benjamini-Hochberg false discovery rate (FDR) correction; protein data in (D-H) analyzed by one-way ANOVA with Tukey post-hoc test. ns – not significant; *p≤0.05; **p≤0.01; ***p≤0.001; ****p≤0.0001.

Abbreviations: WT – wild-type; Thy1-ASO-CNTRL – Thy1-human α-synuclein overexpressing; αSyn – α-synuclein; benCom – beneficial commensal consortium.

Supplementary Figure 2

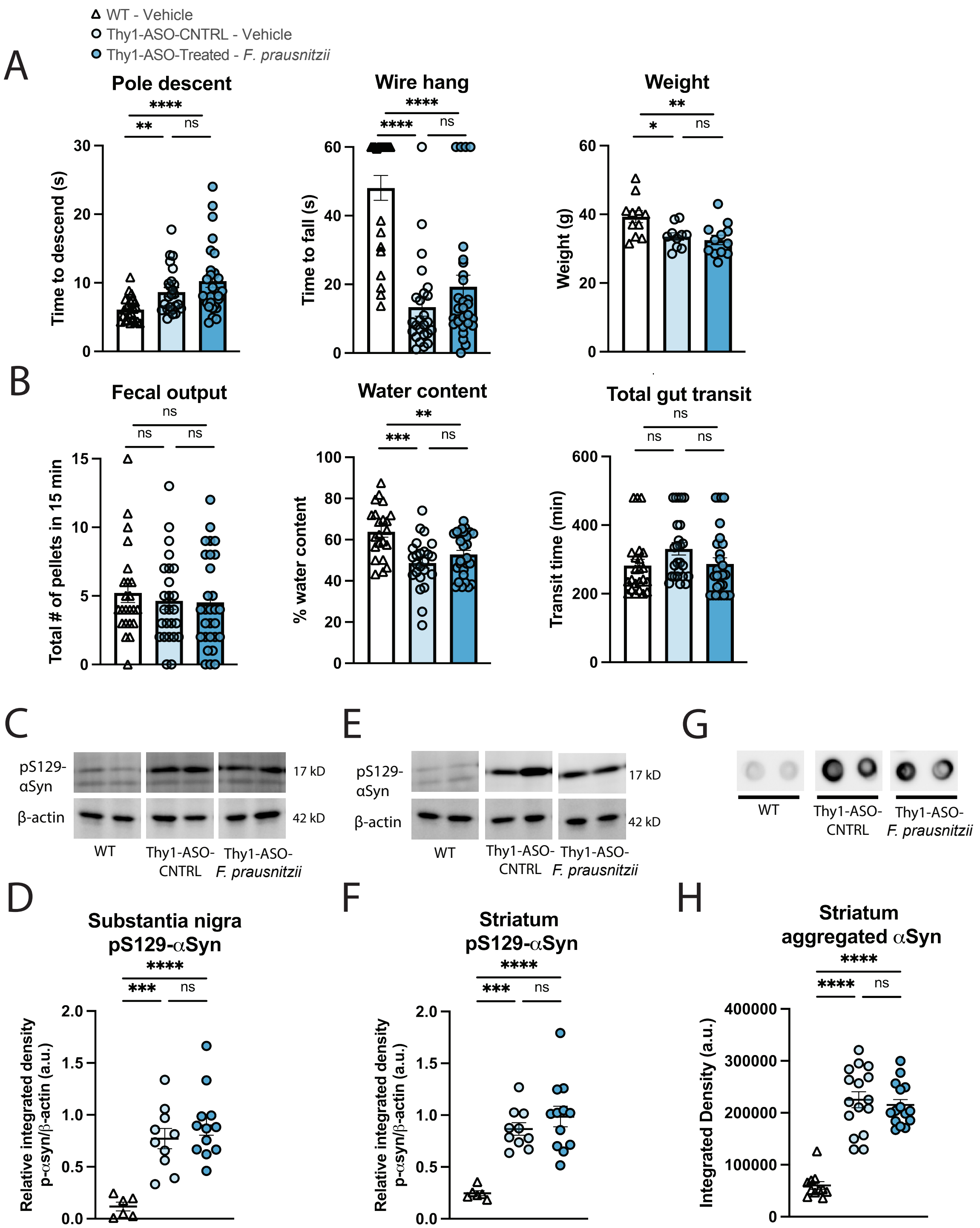

Supplementary Figure 2. Motor and GI function tests and αSyn assays not altered by *F. prausnitzii* treatment. *(Associated with Figure 2)* (**A**) Motor function and animal weights, and (**B**) GI function for *F. prausnitzii*-treated Thy1-ASO animals and controls tested at 20 weeks. n =

23-29. Points represent individual animals, compiled from two independent cohorts; bars represent standard error of the mean. (**C-F**) Representative images (C, E) and quantification (D, F) of Western blots for phosphorylated-S129-α-synuclein in (C, D) substantia nigra and (E, F) striatum. n = 6-12. (**G, H**). Representative images (G) of dot blots and quantification (H) for aggregated α-synuclein in the striatum. n = 11-16. Points represent individual animals, compiled from two independent cohorts; bars represent standard error of the mean. See Supplementary Fig. 11 for uncropped blot images. Behavior data in (A, B) analyzed by Kruskal-Wallis test followed by the Conover-Iman post-hoc test with Benjamini-Hochberg false discovery rate (FDR) correction; protein data in (C-H) analyzed by one-way ANOVA with Tukey post-hoc test. ns – not significant; *$p \leq 0.05$; **$p \leq 0.01$; ***$p \leq 0.001$; ****$p \leq 0.0001$.

Abbreviations: WT – wild-type, Thy1-ASO-CNTRL – Thy1-human α-synuclein overexpressing control; pS129 αSyn – phosphorylated-S129-α-synuclein; αSyn – α-synuclein

Supplementary Figure 3

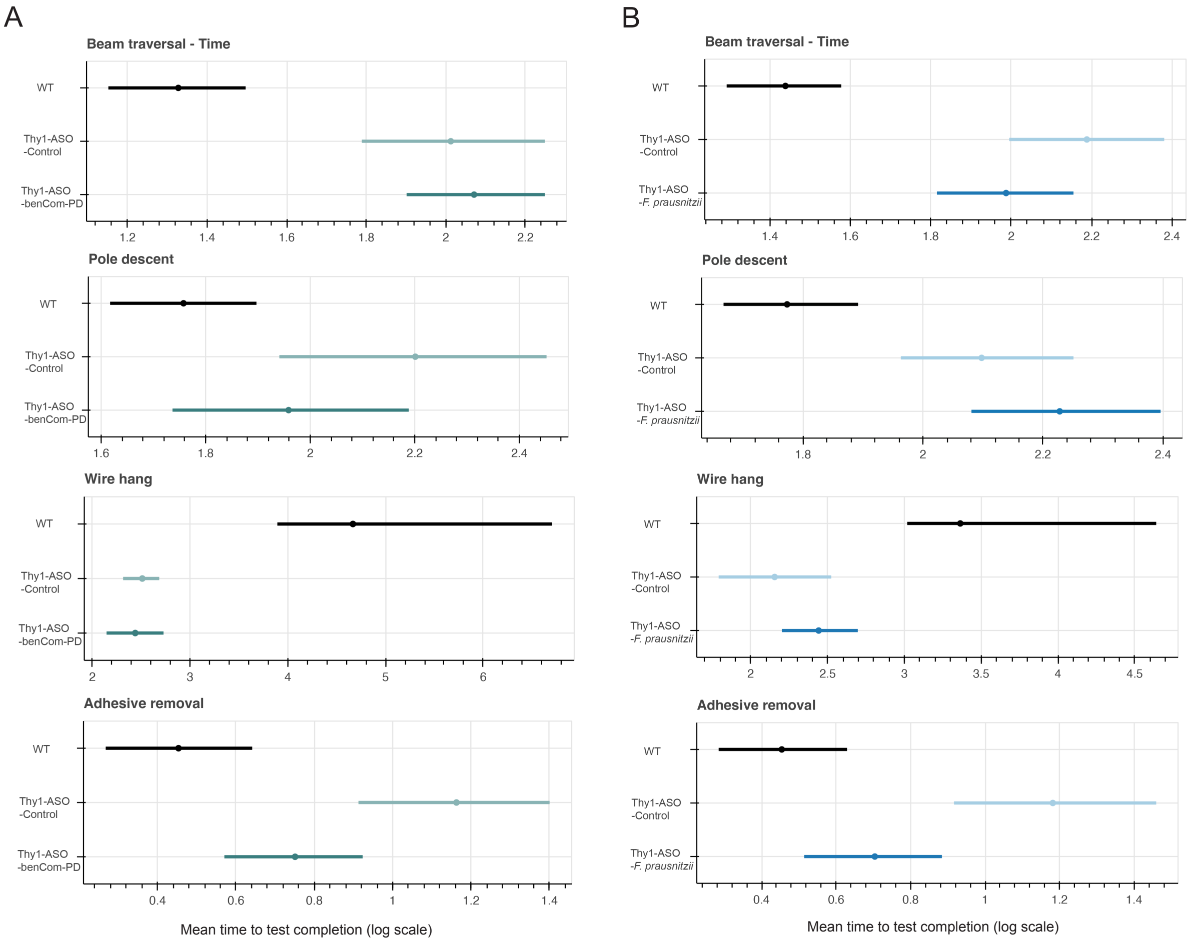

Supplementary Figure 3. Parametric inference analysis of motor function tasks. *(Associated with Figure 2)* Performance of mice treated with (**A**) benCom-PD and (**B**) *F. prausnitzii* was analyzed using generative statistical models that account for ceilings in task completion. Data consisted of individual animals from a single cohort (A) or two independent cohorts (B). Confidence intervals (95% CI) were calculated using maximum likelihood estimation (MLE) followed by parametric bootstrap. The μ 95% CI parameter describes the mean test completion time displayed on a logarithmic scale.

Abbreviations: WT – wild-type, Thy1-ASO – Thy1-human α-synuclein overexpressing

Supplementary Figure 4

A

Test	Group	Q	I² (%)
Beam traversal - Time	Thy1-ASO-Cntrl vs.-Fp	0.426508	0
	WT vs. Thy1-ASO-Fp	1.067422	6.316368
	WT vs. Thy1-ASO-Cntrl	0.121443	0
Beam traversal – Errors per step	Thy1-ASO-Cntrl vs.-Fp	1.961784	49.02599
	WT vs. Thy1-ASO-Fp	0.004704	0
	WT vs. Thy1-ASO-Cntrl	undefined	0
Pole descent	Thy1-ASO-Cntrl vs.-Fp	1.038982	3.751952
	WT vs. Thy1-ASO-Fp	0.068595	0
	WT vs. Thy1-ASO-Cntrl	0.65088	0
Wire hang	Thy1-ASO-Cntrl vs.-Fp	0.00241	0
	WT vs. Thy1-ASO-Fp	0.249885	0
	WT vs. Thy1-ASO-Cntrl	0.997635	0
Adhesive removal	Thy1-ASO-Cntrl vs.-Fp	0.008109	0
	WT vs. Thy1-ASO-Fp	0.008606	0
	WT vs. Thy1-ASO-Cntrl	1.11974	0
Hindlimb clasp	Thy1-ASO-Cntrl vs.-Fp	0.458192	0
	WT vs. Thy1-ASO-Fp	3.418827	70.750201
	WT vs. Thy1-ASO-Cntrl	0.614324	0

B

Test	Group	Q	I² (%)
Fecal output	Thy1-ASO-Cntrl vs.-Fp	1.698515	41.125055
	WT vs. Thy1-ASO-Fp	0.179903	0
	WT vs. Thy1-ASO-Cntrl	1.475329	32.218513
Fecal score	Thy1-ASO-Cntrl vs.-Fp	4.719061	78.809343
	WT vs. Thy1-ASO-Fp	1.506637	33.627016
	WT vs. Thy1-ASO-Cntrl	6.423796	84.432881
Water content	Thy1-ASO-Cntrl vs.-Fp	0.91584	0
	WT vs. Thy1-ASO-Fp	3.229106	69.031675
	WT vs. Thy1-ASO-Cntrl	3.791861	73.627726
Total gut transit	Thy1-ASO-Cntrl vs.-Fp	6.398381	84.371047
	WT vs. Thy1-ASO-Fp	5.014129	80.056356
	WT vs. Thy1-ASO-Cntrl	0.862981	0
Bead expulsion	Thy1-ASO-Cntrl vs.-Fp	1.503285	33.479015
	WT vs. Thy1-ASO-Fp	0.00756	0
	WT vs. Thy1-ASO-Cntrl	3.490956	71.354553

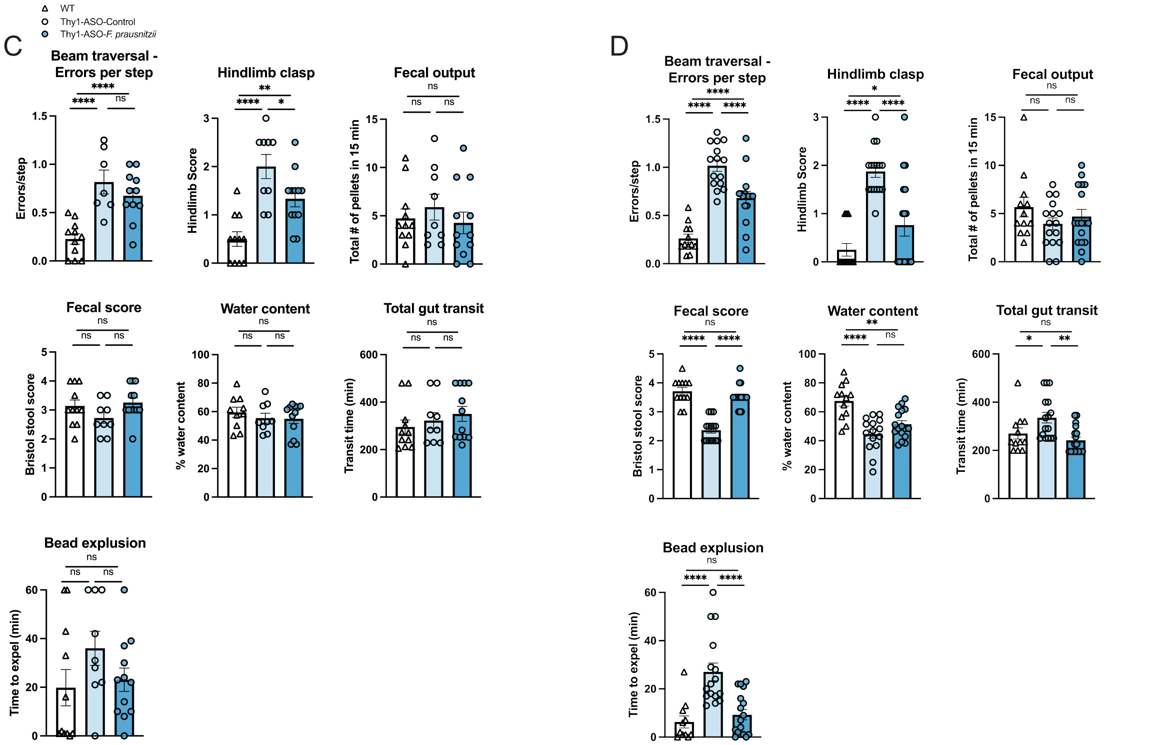

Supplementary Figure 4. Analysis of heterogeneity across cohorts for motor and GI function assays in animals treated with *F. prausnitzii*. *(Associated with Figure 2)* **(A, B)** Variability between cohorts, quantified by Cochran's Q and I^2 statistics, for pairwise comparisons of (A) motor and (B) gut physiology tests. An I^2 value ≤ 20% indicates low to moderate heterogeneity. (**C, D**) Individual cohort results for comparisons with higher heterogeneity: cohort 1, (C, n = 7-11) and cohort 2 (D, n = 12-17). Points represent individual

animals; bars represent standard error of the mean. ns – not significant; *p≤0.05; **p≤0.01; ***p ≤0.001; ****p≤0.0001.

Abbreviations: WT – wild-type, Thy1-ASO-CNTRL – Thy1-human α-synuclein overexpressing control; Fp – *F. prausnitzii*

Supplementary Figure 5

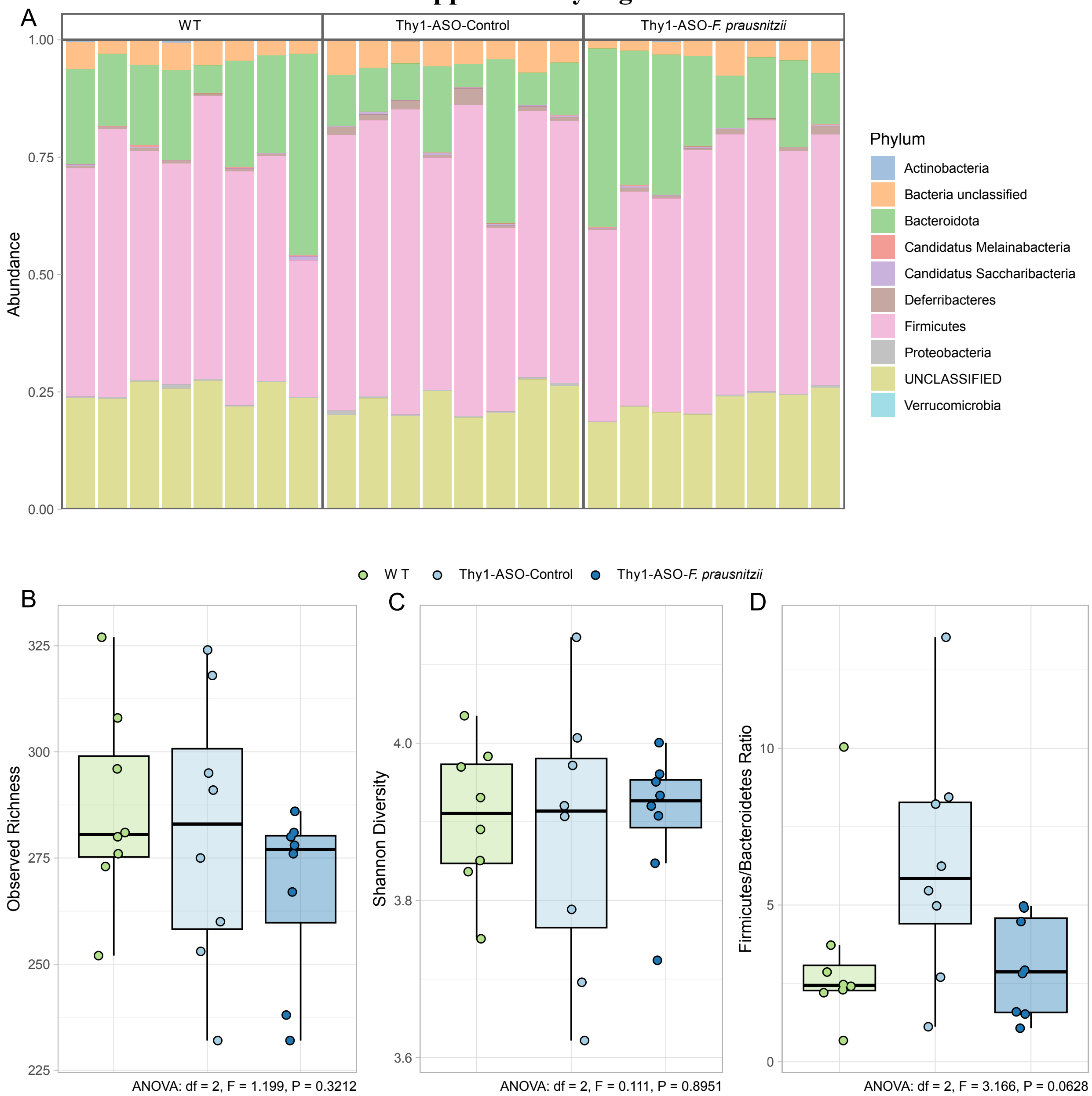

Supplementary Figure 5. Gut microbiome aspects unaltered by *F. prausnitzii* treatment. *(Associated with Figure 3)*. (**A**) Stacked bar plot of relative abundance of bacterial phyla in the gut microbiome, including unclassified reads. Each bar represents a single mouse. (**B-D**) Distributions of (**B**) observed fecal SGB richness, (**C**) Shannon diversity, and (**D**) Firmicutes/Bacteroidetes ratio. Data were analyzed using a one-way ANOVA.

Abbreviations: WT– wild-type; Thy1-ASO – Thy1-human α-synuclein overexpressing; *F. prausnitzii* – *Faecalibacterium prausnitzii;* SGB – Species-level Genome Bin

Supplementary Figure 6

A

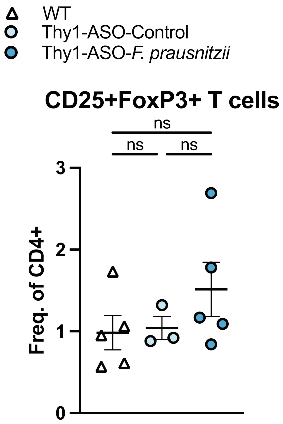

Supplementary Figure 6. T$_{REGS}$ not altered by *F. prausnitzii* supplementation. *(Associated with Figure 4).* (**A**) Quantification of regulatory T cells in mesenteric lymph nodes, expressed as frequency of CD4+ cells. n = 3-5. Points represent individual animals, bars represent standard error of the mean. Data analyzed by one-way ANOVA with Tukey post-hoc test.

Abbreviations: WT – wild-type; Thy1-ASO-CNTRL – Thy1-human α-synuclein overexpressing control; *F. prausnitzii – Faecalibacterium prausnitzii*; ns – not significant

Supplementary Figure 7

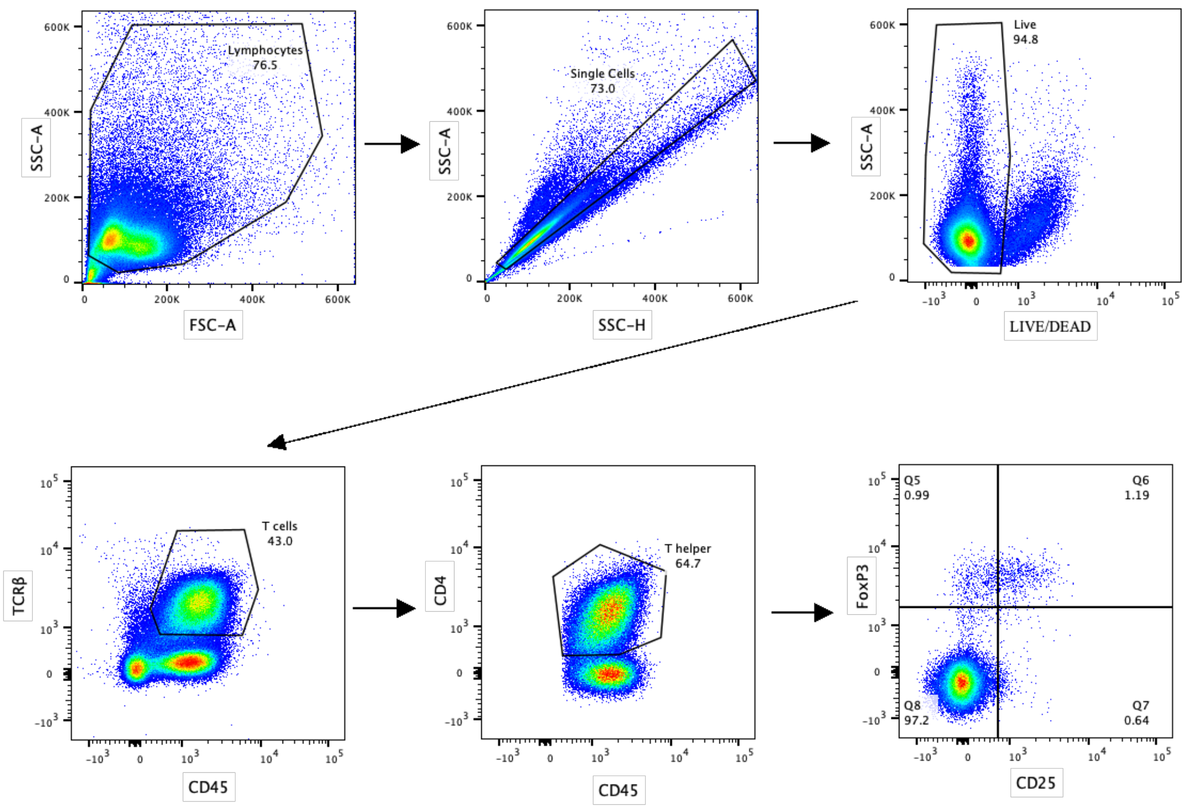

Supplementary Figure 7. Adaptive immune system gating strategy for flow cytometry analysis. *(Associated with Figure 4)*

Supplementary Figure 8

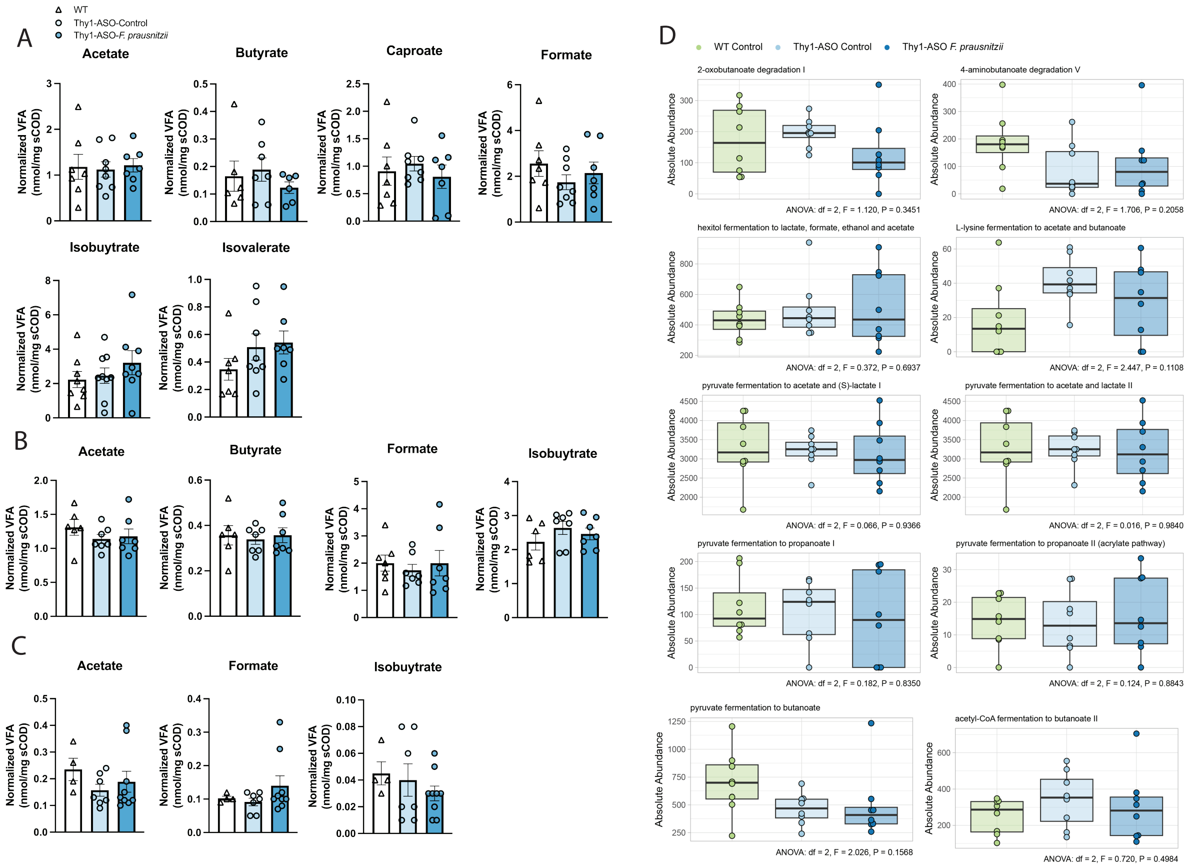

Supplementary Figure 8. Volatile fatty acid and fermentation pathway analyses. (**A**) Fecal, (**B**) cecal, and (**C**) serum detectable volatile fatty acid measurements, normalized by soluble chemical oxygen demand (sCOD), from Thy1-ASO animals treated with *F. prausnitzii* and controls. n = 4-9. Points represent individual animals, bars represent standard error of the mean. Data analyzed by one-way ANOVA with Tukey post-hoc test. No significant changes were detected. Points represent individual animals, bars represent standard error of the mean. (**D**) Boxplots of the abundance of predicted fermentation pathways inferred from metagenomic sequencing data using HUMAnN3. Boxes indicate the median and interquartile range. Data were analyzed using a one-way ANOVA.

Abbreviations: WT – wild-type; Thy1-ASO – Thy1-human α-synuclein overexpressing; *F. prausnitzii* – *Faecalibacterium prausnitzii*

Supplementary Figure 9

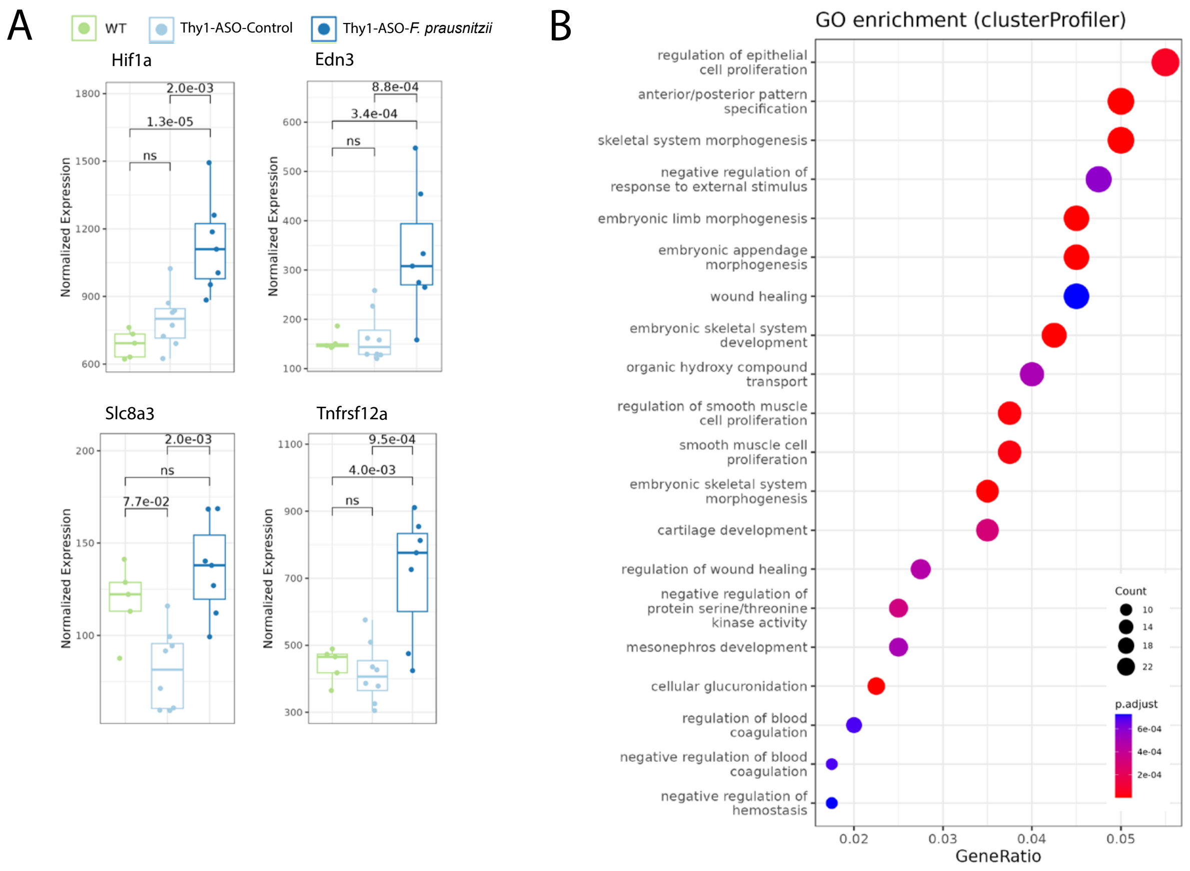

Supplementary Figure 9. RNA sequencing Gene Ontology (GO) enrichment changes following *F. prausnitzii* treatment. *(Associated with Figure 5)* (**A**) Expression levels of differentially regulated immune-related genes of interest found to be involved in GO pathways upregulated by *F. prausnitzii* treatment. Box plots show normalized gene expression values (size-factor scaled counts) with DESeq2 adjusted p-values between comparisons. (**B**) GO enrichment analysis showing pathways downregulated in Thy1-ASO-*F. prausnitzii* tissue v. Thy1-ASO-Control.

Abbreviations: WT – wild-type; Thy1-ASO – Thy1-human α-synuclein overexpressing; *F. prausnitzii* – *Faecalibacterium prausnitzii;* ns – not significant

Supplementary Figure 10

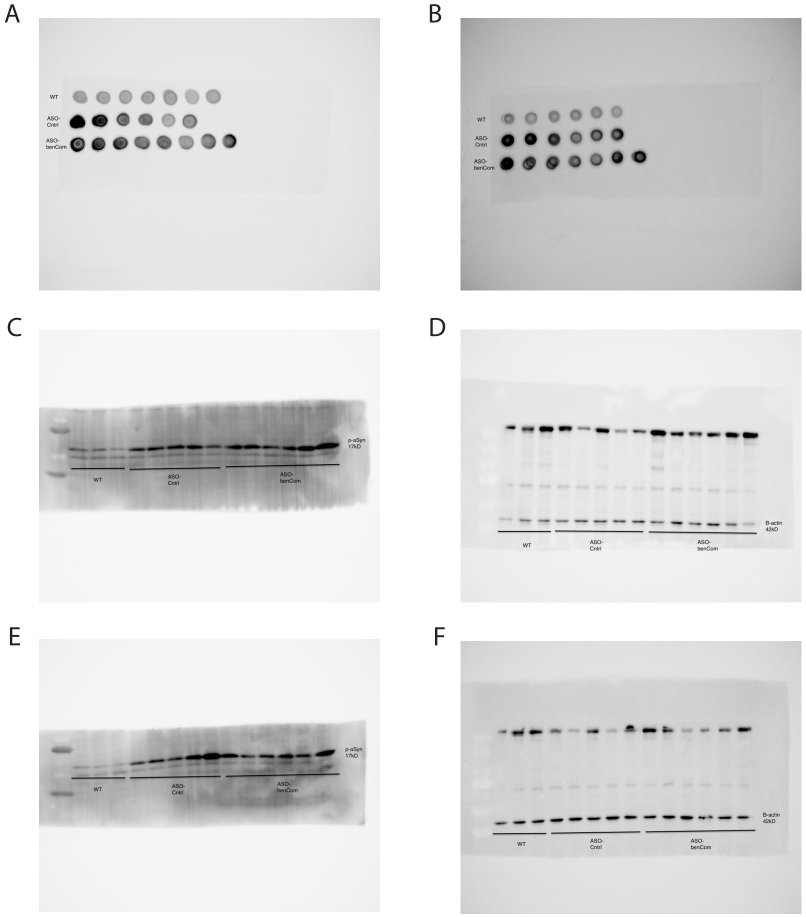

Supplementary Figure 10. Uncropped blot images for benCom-PD experiments. *(Associated with Figure 1 and Supplementary Figure 1)* (**A-B**) Dot blots for aggregated α-synuclein in the substantia nigra (A) and striatum (B) on samples from benCom-PD treated animals and controls. (**C-D**) Western blots for phosphorylated-S129-α-synuclein in the substantia nigra (C) and corresponding β-actin loading control (D). (**E-F**) Western blots for phosphorylated-S129-α-synuclein in the striatum (E) and corresponding β-actin loading control (F).

Abbreviations: WT – wild-type; ASO – Thy1-human α-synuclein overexpressing; Cntrl – Control; benCom – benCom-PD*;* p-αSyn – phosphorylated-S129-α-synuclein

Supplementary Figure 11

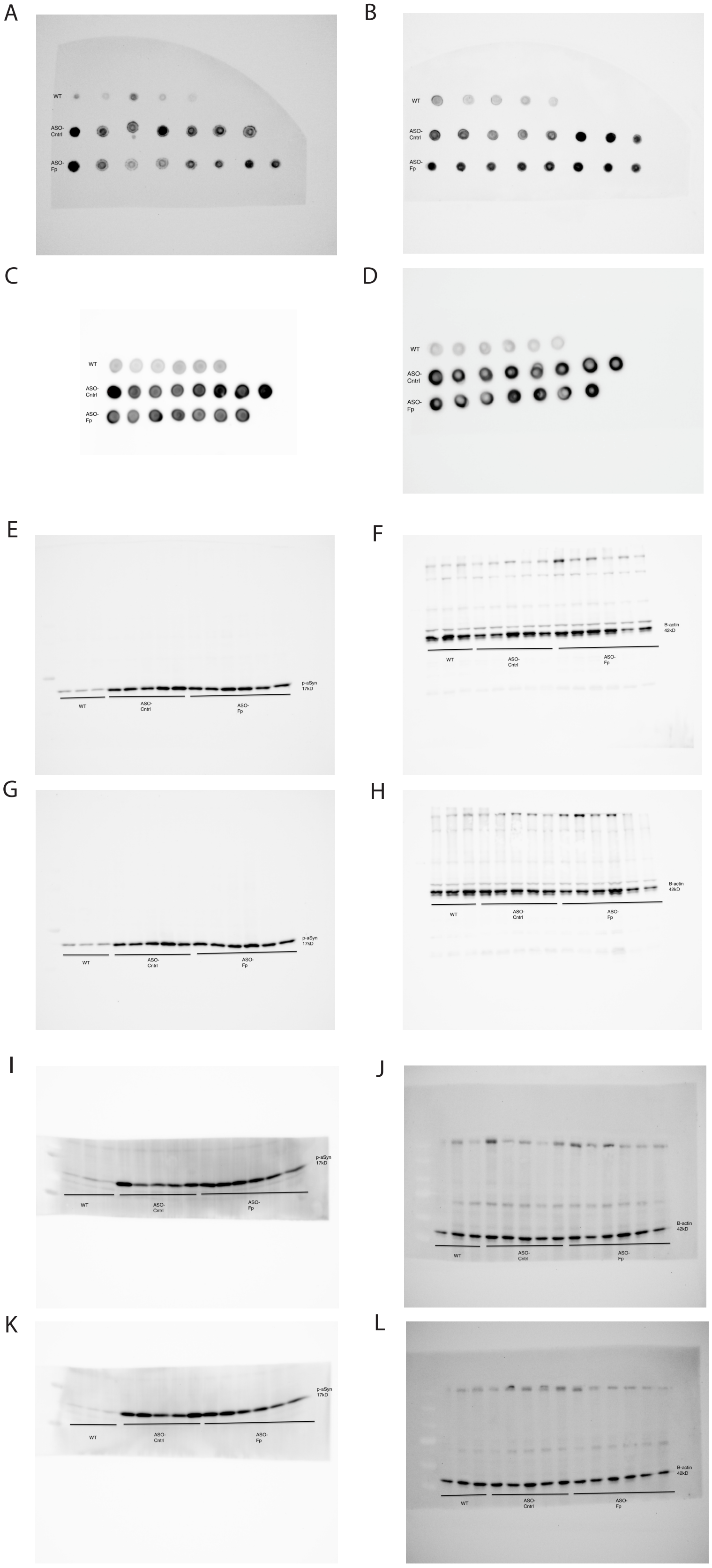

Supplementary Figure 11. Uncropped blot images for *F. prausnitzii* experiments. *(Associated with Figure 2 and Supplementary Figure 2)* (**A-D**) Dot blots for aggregated α-synuclein in the substantia nigra cohort 1 (A) and cohort 2 (C), and striatum cohort 1 (B) and cohort 2 (D) on samples from *F. prausnitzii* treated animals and controls. (**E-H**) Western blots for phosphorylated-S129-α-synuclein in the substantia nigra cohort 1 (E) and cohort 2 (G) and corresponding β-actin loading control for cohort 1 (F) and 2 (H). (**I-L**) Western blots for phosphorylated-S129-α-synuclein in the striatum cohort 1 (I) and cohort 2 (K) and corresponding β-actin loading control for cohort 1 (J) and 2 (L).

Abbreviations: WT – wild-type; ASO – Thy1-human α-synuclein overexpressing; Cntrl – Control; Fp – *Faecalibacterium prausnitzii;* p-αSyn – phosphorylated-S129-α-synuclein

Supplementary Table 1

Taxon Name	Taxonomic Level	Total signatures	Increased signatures	Decreased signatures
Akkermansia	genus	39	39	0
Faecalibacterium	genus	47	4	43
Roseburia	genus	46	4	42
Bifidobacterium	genus	37	34	3
Blautia	genus	26	2	24
Faecalibacterium prausnitzii	species	16	0	16
Fusicatenibacter	genus	20	1	19
Roseburia intestinalis	species	12	0	12
Blautia wexlerae	species	9	0	9
Roseburia faecis	species	7	0	7
Prevotella	genus	26	10	16
Anaerostipes	genus	8	3	5
Eubacterium	genus	7	3	4

Supplementary Table 1. **Summary of microbial signatures across human Parkinson's disease studies.** Taxa found to be altered at the genus and species levels in human PD gut microbiome as of December 14, 2025, using BugSigDB (https://bugsigdb.org/; Geistlinger et al., 2024), a comprehensive database of published microbial signatures. Refer to **Supplementary Data 1** for a full list of the Top 80 signatures.

Supplementary Table 2. Key Resource Table
Data, code, protocols, and key lab materials used and generated in this study.

RESOURCE TYPE	RESOURCE NAME	SOURCE	IDENTIFIER	NEW/ REUSE	ADDITIONAL INFORMATION
Antibody	Anti-phosphorylated S129 a-Synuclein Rabbit	abcam	ab51253; RRID:AB_869973	Reuse	1:1000 concentration
Antibody	Anti-β-actin Mouse	abcam	ab8226; RRID:AB_306371	Reuse	1:1000 concentration
Antibody	Anti-IgG HRP Rabbit	Cell Signaling Technology	7074; RRID:AB_2099233	Reuse	1:1000 concentration
Antibody	Anti-IgG HRP Mouse	Cell Signaling Technology	7076; RRID:AB_330924	Reuse	1:1000 concentration
Antibody	Anti-Aggregated a-Synuclein Rabbit	abcam	ab209538; RRID:AB_2714215	Reuse	1:1000 concentration
Antibody	anti-Fcγ receptor (anti-CD16/CD32) (clone 93)	eBioscience	14-0161-86; RRID:AB_467135	Reuse	1:100 concentration
Antibody	CD45 (clone 30-F11)	eBioscience	48-0451-82; RRID:AB_1518806	Reuse	1:500 concentration
Antibody	TCRB (clone H57-597)	eBioscience	45-5961-82; RRID:AB_925763	Reuse	1:500 concentration
Antibody	CD4 (clone RM4-5)	eBioscience	25-0042-82; RRID:AB_469578	Reuse	1:500 concentration
Antibody	CD25 (clone PC61.5)	eBioscience	17-0251-82; RRID:AB_469366	Reuse	1:500 concentration
Antibody	FoxP3 (clone FJK-16s)	eBioscience	53-5773-82; RRID:AB_763537	Reuse	1:100 concentration
Bacterial strain	*Roseburia intestinalis*	DSMZ	DSMZ14610	Reuse	
Bacterial strain	*Roseburia faecis*	DSMZ	DSMZ16840	Reuse	
Bacterial strain	*Anaerostipes hadrus*	ATCC	ATCC29173	Reuse	
Bacterial strain	*Fusicatenibacter saccharivorans*	DSMZ	DSMZ26063	Reuse	
Bacterial strain	*Prevotella histicola*	DSMZ	DSMZ19854	Reuse	
Bacterial strain	*Bacteroides ovatus*	ATCC	ATCC8483	Reuse	
Bacterial strain	*Eubacterium rectale*	ATCC	ATCC33656	Reuse	
Bacterial strain	*Faecalibacterium prausnitzii*	DSMZ	DSMZ17677	Reuse	

Chemical, peptide, or recombinant protein	Carmine	Millipore Sigma	C1022	Reuse	
Chemical, peptide, or recombinant protein	Phorbol Myristate Acetate (PMA)	Sigma Aldrich	P1585	Reuse	50 ng/ml
Chemical, peptide, or recombinant protein	Ionomycin	Sigma Aldrich	I0634	Reuse	750 ng/ml
Critical commercial assay	Pierce BCA Protein Assay Kit	Thermo Scientific	23225	Reuse	
Critical commercial assay	Bio-Plex Pro Mouse Th17 6-plex Assay	Bio-Rad	M6000007NY	Reuse	
Critical commercial assay	Direct-zol RNA MiniPrep Plus Kit	Zymo Research	R2072	Reuse	
Critical commercial assay	FAST RNA-seq Lib Prep Kit (v2)	ABclonal	RK20306	Reuse	
Critical commercial assay	Poly(A) mRNA Capture Module	ABclonal	RK20340	Reuse	
Critical commercial assay	High-range (20 – 1500 mg COD/L) COD kit	Hach	2565115	Reuse	
Critical commercial assay	QIAamp PowerFecal Pro kit	Qiagen	51804	Reuse	
Critical commercial assay	Quant-iT™ 1X dsDNA Assay Kit, BR	Life Technologies	Q33267	Reuse	
Critical commercial assay	Illumina DNA Prep, (M) Tagmentation (96 Samples, IPB) kit	Illumina	20060059	Reuse	
Dataset	Beam Traversal Videos	Github/Zenodo	https://doi.org/10.5281/zenodo.17137678	New	
Dataset	Motor and GI function data	Github/Zenodo	https://doi.org/10.5281/zenodo.17137678	New	
Dataset	Blot images & values	Github/Zenodo	https://doi.org/10.5281/zenodo.17137678	New	
Dataset	Protein assays data	Github/Zenodo	https://doi.org/10.5281/zenodo.17137678	New	
Dataset	Flow cytometry data	Zenodo	https://doi.org/10.5281/zenodo.16929959	New	
Dataset	RNAseq data	SRA	PRJNA1308739	New	

Dataset	Volatile fatty acid analysis data	Github/Zenodo	https://doi.org/10.5281/zenodo.17137678	New	
Dataset	Microbiome sequencing data	SRA	PRJNA1259538	New	
Experimental model: Organism/ strain	Thy1-human α-synuclein overexpressing mice "Line 61"	Masliah Lab	RRID: IMSR_JAX:038796	Reuse	Chesselet et al. Neurother 9, 297–314 (2012); Rockenstein et al. J Neurosci Res 68:568–578 (2002)
Other	Yeast Casitone Fatty Acids with Carbohydrates (YCFAC) media	Anaerobe Systems	AS-680	Reuse	
Other	Peptone Yeast Extract Broth with Glucose (PYG) media	Anaerobe Systems	AS-822	Reuse	
Other	Mega Medium	N/A	N/A	Reuse	Cheng, A. G. *et al.* Design, construction, and in vivo augmentation of a complex gut microbiome. *Cell* 185, 3617-3636.e19 (2022).
Other	Chopped Meat with Carbohydrates (CMC)	Anaerobe Systems	AS-823	Reuse	
Other	AnaeroPack	MGC	#10-01	Reuse	
Other	Bio-Plex Cell Lysis Kit	Bio-Rad	171304011	Reuse	
Other	Novex™ Tris-Glycine Mini protein gels ,4-20%	Invitrogen	XP04205BOX	Reuse	
Other	Immobilon-P 0.45um PVDF membrane	Merck Millipore Ltd.	IPVH00010	Reuse	
Other	Clarity Western ECL Substrate	Bio-Rad	170-5061	Reuse	
Other	Nitrocellulose membrane	Thermo Scientific	88018	Reuse	
Other	Lysing Matrix D Bead beater tubes	MP Biomedicals	6913500	Reuse	
Other	Hybri-Max - Red blood cell lysing buffer	Sigma Aldrich	R7757	Reuse	
Other	GolgiPlugTM	BD Biosciences	555029	Reuse	1:1000 concentration

Other	Fixable Near-IR LIVE/DEAD Stain Kit	Invitrogen	L10119	Reuse	1:500 concentration
Other	Foxp3/Transcription Factor Fixation/Permeabilization Kit	eBioscience	00-5521-00	Reuse	
Other	TRIzol reagent	Invitrogen	15596-026	Reuse	
Other	Sarstedt Z-gel Microtube 1.1ml	Fisher scientific	50-809-211	Reuse	
Other	Aminex HPX-87H column	Bio-Rad	1250095	Reuse	
Other	10 mM VFA mixed standard	Supelco	CRM46975	Reuse	
Protocol	Bacterial cultures	protocols.io	dx.doi.org/10.17504/protocols.io.81wgboqw1lpk/v1	New	
Protocol	Beam traversal test	protocols.io	dx.doi.org/10.17504/protocols.io.kxygx4znzl8j/v1	New	
Protocol	Pole descent test	protocols.io	dx.doi.org/10.17504/protocols.io.8epv5k9mjv1b/v1	New	
Protocol	Wire hang test	protocols.io	dx.doi.org/10.17504/protocols.io.6qpvrw62plmk/v1	New	
Protocol	Adhesive removal test	protocols.io	dx.doi.org/10.17504/protocols.io.4r3l21objg1y/v1	New	
Protocol	Hindlimb clasping test	protocols.io	dx.doi.org/10.17504/protocols.io.eq2ly4n2mlx9/v1	Reuse	
Protocol	Fecal output, score, and water content	protocols.io	dx.doi.org/10.17504/protocols.io.6qpvrw6qplmk/v1	New	
Protocol	Total gut transit test	protocols.io	dx.doi.org/10.17504/protocols.io.rm7vz9yq8gx1/v1	Reuse	
Protocol	Bead expulsion test	protocols.io	dx.doi.org/10.17504/protocols.io.n92ld6zn7g5b/v1	New	
Protocol	Western blot assay	protocols.io	dx.doi.org/10.17504/protocols.io.x54v92z2ql3e/v1	Reuse	
Protocol	Dot blot assay	protocols.io	dx.doi.org/10.17504/protocols.io.261gen2xdg47/v1	Reuse	
Protocol	Immune cell Isolation	protocols.io	dx.doi.org/10.17504/protocols.io.nm2dc8e	Reuse	
Protocol	RNA sequencing	protocols.io	dx.doi.org/10.17504/protocols.io.6qpvry7wogmk/v1	New	
Protocol	Metagenomics sample processing	protocols.io	dx.doi.org/10.17504/protocols.io.e6nvw15dwlmk/v1	Reuse	
Protocol	VFA measurement	protocols.io	dx.doi.org/10.17504/protocols.io.e6nvw4wo7lmk/v1	Reuse	
Software/code	Python	python.org	https://www.python.org/, RRID:SCR_008394	Reuse	

Software/code	FIJI v2.10.0	National Institute of Health (NIH)	https://doi.og/10.1038/nmeth.2019; RRID: SCR_002285	Reuse	
Software/code	FlowJo v11	BD, Tree Star	https://www.flowjo.com/solutions/flowjo; RRID:SCR_008520	Reuse	
Software/code	R code: RNAseq Analysis script	Github/Zenodo	https://doi.org/10.5281/zenodo.17981510	New	
Software/code	GraphPad Prism 10	GraphPad Software/ Dotmatics	http://www.graphpad.com; RRID:SCR_002798	Reuse	
Software/code	R code: Microbiome sequencing analysis script	Github/Zenodo	https://doi.org/10.5281/zenodo.17128213	New	
Software/code	STAR aligner v2.7.1	Github/Zenodo	https://doi.org/10.1093/bioinformatics/bts635, RRID:SCR_004463	Reuse	
Software/code	featureCounts	Bioconductor	https://subread.sourceforge.net/featureCounts.html, RRID:SCR_012919	Reuse	
Software/code	DESeq2 package v1.50.2	Github/Zenodo	https://doi.org/10.18129/B9.bioc.DESeq2 RRID:SCR_015687	Reuse	
Software/code	R v4.3.0	R project	http://www.r-project.org; RRID:SCR_001905	Reuse	
Software/code	clusterProfiler package_v4.18.2	Bioconductor	https://doi.org/10.18129/B9.bioc.clusterProfiler; RRID:SCR_016884	Reuse	
Software/code	ggplot2 v4.0.1	CRAN	https://doi.org/10.32614/CRAN.package.ggplot2; RRID:SCR_014601	Reuse	
Software/code	MetaPhlAn v4.1.1	Huttenhower Lab	https://doi.org/10.1038/s41587-023-01688-w; RRID:SCR_004915	Reuse	
Software/code	HUMANnN v3.9	Huttenhower Lab	https://doi.org/10.7554/eLife.65088 ; RRID:SCR_014620	Reuse	
Software/code	mia package v1.18.0	Bioconductor	https://doi.org/10.18129/B9.bioc.mia; RRID:SCR_023619	Reuse	
Software/code	MaAsLin3	Huttenhower Lab	https://huttenhower.sph.harvard.edu/maaslin/, RRID:SCR_023241	Reuse	
Software/code	“mediation” R package v4.5.1	CRAN	https://doi.org/10.32614/CRAN.package.mediation; RRID:SCR_026984	Reuse	
Software/code	BioRender	BioRender	https://BioRender.com/zvkyy6g, RRID: SCR_018361	Reuse	
